# The bacterial community composition of the surface microlayer in a high mountain lake

**DOI:** 10.1111/j.1574-6941.2010.00904.x

**Published:** 2010-09

**Authors:** Paul Hörtnagl, Maria Teresa Pérez, Michael Zeder, Ruben Sommaruga

**Affiliations:** 1 Laboratory of Aquatic Photobiology and Plankton Ecology, Institute of Ecology, University of InnsbruckInnsbruck, Austria; 2 Department of Limnology, Institute of Plant Biology, University of ZürichKilchberg, Switzerland

**Keywords:** air–water interface, bacterioneuston, bacterial seasonality, *Betaproteobacteria*, UV radiation, CARD-FISH, alpine lake

## Abstract

The existence of bacterioneuston in aquatic ecosystems is well established, but little is known about its composition and dynamics, particularly in lakes. The bacterioneuston underlies extreme conditions at the air–water boundary, which may influence its dynamics in a different way compared with the bacterioplankton. In this study, we assessed quantitative changes in major bacterial groups of the surface microlayer (SML) (upper 900 μm) and the underlying water (ULW) (0.2–0.5 m depth) of an alpine lake during two consecutive ice-free seasons. Analysis of the bacterial community composition was done using catalyzed reporter deposition FISH with oligonucleotide probes. In addition, several physicochemical parameters were measured to characterize these two water layers. Dissolved organic carbon was consistently enriched in the SML and the dissolved organic matter pool presented clear signals of photodegradation and photobleaching. The water temperature was generally colder in the SML than in the subsurface. The bacterial community of the SML and the ULW was dominated by *Betaproteobacteria* and *Actinobacteria*. The bacterial community composition was associated with different combinations of physicochemical factors in these two layers, but temporal changes showed similar trends in both layers over the two seasons. Our results identify the SML of alpine lakes as a microhabitat where specific bacterial members such as of *Betaproteobacteria* seem to be efficient colonizers.

## Introduction

In marine and freshwater ecosystems, the air–water boundary, known as the surface microlayer (SML), constitutes an important interface between the troposphere and the underlying water (ULW). Because of its enormous area, processes taking place at the SML such as gas exchange between the hydrosphere and the atmosphere are of fundamental relevance for the biosphere (Conrad & Seiler, 1988; Upstill-Goddard *et al.*, 2003; Asher, 2005;). Understanding the composition and the dynamics of the microbial community present at the SML is crucial because microorganisms are known to play key roles in many biogeochemical processes at this interface (Sieburth, 1983; Conrad & Seiler, 1988; Upstill-Goddard *et al.*, 2003; Agogué*et al.*, 2005;). The SML of both marine and freshwater ecosystems is usually enriched in organic and inorganic nutrients (Maki, 1993; Liss & Duce, 2005;). Especially in oligotrophic systems, enrichments in nutrients could be favorable for bacteria inhabiting the SML (Sieburth *et al.*, 1976).

In contrast to the knowledge on the physicochemical properties of the SML, information on its biological characteristics is still scarce. Although several studies have compared bacterial abundance (Agogué*et al.*, 2005; Joux *et al.*, 2006; Cunliffe *et al.*, 2009c;) and bacterial activity (Hermansson & Dahlbäck, 1983; Hörtnagl *et al.*, 2010;) between bacterioneuston (i.e. bacteria inhabiting the SML) and bacterioplankton, knowledge of the composition of the bacterioneuston is still limited, particularly in lakes. Previous studies have been restricted to descriptive and isolating approaches (Fehon & Oliver, 1979; Nikitin *et al.*, 2004; Pladdies *et al.*, 2004;), and only very few studies have directly compared the bacterial community composition of the SML and the ULW using cultivation-independent methods (Franklin *et al.*, 2005; Auguet & Casamayor, 2008; Cunliffe *et al.*, 2009a,c; Hervàs & Casamayor, 2009;). Furthermore, available information refers to comparisons based on single sampling efforts, but data on temporal changes of freshwater bacterioneuston are not available to the best of our knowledge.

Compared with lowland lakes, high mountain lakes (i.e. located above the treeline) are harsh environments even for bacteria because these ecosystems undergo relatively rapid changes in environmental conditions such as water temperature, light, UV exposure, and nutrient concentrations. Lakes located at a high altitude are very sensitive to environmental changes and thus particularly interesting with regard to potential changes in the bacterial community composition. Previous studies have shown that the bacterial community composition of high mountain lakes differs among different water layers. For example, Alfreider *et al.* (1996) found differences in the bacterial community composition of snow, slush, and lake water layers in a high mountain lake of the Alps, Austria, and Hervàs & Casamayor (2009) found qualitative differences in the bacterial community composition of the SML and the ULW of a high mountain lake in the Pyrenees, Spain. The latter authors also found a high similarity between the composition of the bacterioneuston and the air-borne bacteria deposited on top of the snow cover of the lake. It remains unknown, however, whether the bacterial assemblage of the SML can be defined as ‘distinct’ compared with the ULW and how variable bacterioneuston composition is over long periods. Moreover, we know little about what factors influence the bacterial community structure in the SML of lakes. Because of the extreme conditions found at the SML and the direct influence of air-borne bacteria on this boundary, we hypothesized that the conditions influencing the composition of the bacterioneuston and the bacterioplankton are different. Thus, the aim of the present study was to determine the composition of bacterioneuston from an alpine lake as well as to identify linkages between the bacterial community composition and key environmental parameters. For this purpose, we followed quantitative changes in the bacterial community composition and in the physicochemical characteristics of the SML and compared them with the ULW during two consecutive ice-free periods.

## Material and methods

### Study site and sampling

For this study, we selected the small alpine lake Gossenköllesee (GKS; area: 0.017 km^2^) that is located at 2417 m above sea level in the Austrian Alps (47°13′N, 11°01′E). Gossenköllesee is a transparent dimictic and holomictic lake covered by ice for about 7–8 months per year. The lake is embedded into a steep catchment area (about 0.3 km^2^) consisting of crystalline rocks that surround the lake northwards and rise approximately 350 m above the water surface. Southwards, the lake is surrounded only by a small mound in the south-west, so that the lake generally appears to be south exposed. Sampling was performed at around noon under stable weather conditions (neither rain nor wind velocities higher than 5 m s^−1^). Samples from the SML and the ULW were collected at the center of the lake from a boat at biweekly intervals during two consecutive ice-free seasons (from June to October) in 2007 and 2008. The SML was sampled using a modified screen sampler (Agogué*et al.*, 2004) consisting of an acid-stable plastic frame with a fixed aluminum screen (mesh size of 1 mm). The screen sampler was laid horizontally on the lake surface and water was collected from the upper 900 μm (Hörtnagl *et al.*, 2010). To include possible spatial heterogeneity of the SML and to obtain enough volume for all analyses, the sampler was used at least 15 times at randomly selected points of the lake surface in the central area. Before every sampling event, the screen sampler was washed with diluted hydrochloric acid and rinsed extensively with Milli-Q water several times to avoid organic contamination. The ULW was sampled with a 5-L bottle sampler (Uwitec, Mondsee, Austria) that included the water layer between 0.2 and 0.5 m depth. All samples were transported within 4 h to the laboratory at the *in situ* temperature.

### Physicochemical parameters

The water temperature of both layers was measured using a glass thermometer (±0.1 °C) immediately after sample collection, and the wind velocity was measured using an HP 816 anemometer (TMT-Top Messtechnik, Erdnig, Germany) at the shore 2 m above the surface. Samples for the optical characterization of dissolved organic matter (DOM) and for the estimation of the dissolved organic carbon (DOC) and total dissolved organic nitrogen (TDN) concentration were directly collected into precombusted (4 h at 450 °C) glass bottles with glass stoppers (100 mL, Schott). Samples for pH and total dissolved phosphorous (TDP) were filled into polyethylene bottles of 1 L (HCl-cleaned and rinsed several times with Milli-Q and sample water). At the laboratory (*c*. 4 h after collection), samples for chemical parameters were filtered through a precombusted (450 °C for 2 h) glass fiber filter (Whatman, GF/F). The filtered water sample was stored at 4 °C in the dark until further analysis within 24 h.

Conductivity was measured using an LF196 sensor (WTW, Weilheim, Germany) and pH using an Orion 960 meter (Thermo Fischer Scientific, Waltham, MA). Samples for DOC and TDN analyses were filtered using a stainless-steel syringe holder with two precombusted (2 h at 450 °C) glass fiber filters (Whatman, GF/F) that were exchanged for each sample. Both the syringe holder and the precombusted filters were rinsed previously with 20 mL of Milli-Q water and 10 mL of the sample. The filtrate was collected in precombusted (4 h at 450 °C) glass vials (40 mL, Shimadzu), then acidified with HCl to pH 2, and stored at 4 °C in the dark until further processing (within 24 h). DOC and TDN were analyzed using a total organic carbon analyzer (Shimadzu TOC-Vc series) equipped with a total nitrogen (TNM-1) module. For the DOC analysis, the instrument was calibrated with potassium hydrogen phthalate in the range of 0.4–4 mg L^−1^, whereas for the analysis of TDN, potassium nitrate in the range of 0.1–2 mg L^−1^ was used. Three to five subsamples were analyzed for each sample and for a consensus reference material (CRM) for DOC (batch 5 FS-2005: 0.57 mg C L^−1^; provided by RSMAS/MAC, University of Miami) that was run in parallel on each occasion. Results differed from the CRM given value by 5%, and the coefficient of variation among subsamples was <2%.

The optical characterization of DOM was performed spectrophotometrically. First, the sample was filtered (the same filtration procedure as that described for DOC and TDN analyses) into a 10-cm quartz cuvette (SUPRASIL I) and then scanned in a double-beam spectrophotometer (Hitachi U2001) between 250 and 750 nm (reference: Milli-Q water). The absorption coefficient at a specific wavelength (abs_λ_) was calculated using the formula abs_λ_=(*D*_λ_× ln 10)/*L*, where *D*_λ_ is the absorbance at the considered wavelength and *L* is the path length (m) of the cuvette. The absolute absorption coefficient (*a*_λ_) was calculated after correcting for the absorption at 750 nm to account for potential scattering of small particles. The dominant relative molecular size of DOM was assessed by calculating the ratio between the absorption at 254 and 365 nm (Strome & Miller, 1978; DeHaan, 1993;). Additionally, the DOC-specific UV absorption at 254 nm (SUVA) was used as a proxy for the degree of aromaticity of DOM (Weishaar *et al.*, 2003).

The concentration of TDP was determined by spectrophotometry following the molybdate method after digestion of the sample with sulfuric acid and hydrogen peroxide (Schmid & Ambühl, 1965).

### Bacterial parameters

Samples for bacterial abundance were fixed immediately after sampling with formaldehyde (2% final concentration) and stored at 4 °C in the laboratory. On the next day, 15 mL of the sample was filtered onto black polycarbonate filters (Millipore, Type GBTP, 0.22 μm) and stained with DAPI (4′,6-diamidino-2-phenylindole, Molecular Probes, Eugene, OR) according to the method described by Porter & Feig (1980). An epifluorescence microscope (Axiophot, Carl Zeiss) equipped with a filter set for DAPI (Filter set no. 1) was used to count at least 500 cells in more than 10 microscopic fields per filter.

The composition of the bacterial community was assessed using the catalyzed reporter deposition FISH (CARD-FISH) method according to Pernthaler *et al.* (2002). For this purpose, a sample of 15 mL was fixed with formaldehyde (2% final concentration) and then filtered onto a white polycarbonate filter (Millipore, Type GTTP, 0.22 μm). The filters were stored at −20 °C until further processing within 1 month. Thawed filters were embedded in low-gelling point agarose (0.2%) and permeabilized according to the protocol of Sekar *et al.* (2003). Detection of major freshwater bacterial groups was performed with six group-specific 5′-horseradish peroxidase-labeled oligonucleotide probes (Thermo-Hybaid, Germany) targeting *Bacteria* (EUBI-III) (Daims *et al.*, 1999), *Alphaproteobacteria* (ALF968) (Neef, 1997), *Betaproteobacteria* (BET42a) (Manz *et al.*, 1992), the R-BT subgroup of the *Betaproteobacteria* (R-BT065) (Šimek *et al.*, 2001), *Actinobacteria* (HGC69a) (Roller *et al.*, 1994), and ‘*Cytophaga*-like’ (CF319a) (Manz *et al.*, 1996; Kirchman, 2002;). The probe CF319a targets members of the phylum *Bacteroidetes*, but it was designed to target most of the *Flavobacteria* and *Sphingobacteria* (90%). The specificity of the probes used in this study has been assessed recently (Amann & Fuchs, 2008). Except for the probe CF319a, the group coverage of the other probes is high (>80%) and outgroup hits yield bacteria that are typically not found in unpolluted freshwaters, such as members of the *Gamma proteobacteria* (Amann & Fuchs, 2008). Archaea were not included in this study because they are not numerically important in the bacterioneuston of this lake in summer (Hörtnagl *et al.*, 2010) or in the bacterioplankton (Pernthaler *et al.*, 1998).

The formamide concentration in the hybridization buffer was 55%, except for the probe HGC69a (35% formamide concentration). All hybridizations were run for 5 h at 35 °C, followed by 30 min of signal-amplification performed in a 0.5-mL reaction vial containing amplification buffer and tyramide-Alexa 488 (Invitrogen Ltd, Paisley, UK) in a 1 : 100 ratio. The majority of the CARD-FISH preparations (about 80%) were evaluated by automated cell counting. A detailed description of the automated evaluation process can be found elsewhere (Zeder *et al.*, 2009). Other preparations (about 20%) were counted manually using a Zeiss Axioplan microscope equipped with a Hg lamp (HBO 100) and the Zeiss filter sets for DAPI and Alexa488 (Filter sets: 1 and 9, respectively). More than 2000 DAPI-positive cells for the automated evaluation process and at least 400 DAPI-positive cells for the manual evaluation process were counted in more than 20 different randomly selected microscopic fields. Both evaluation processes were compared with one another for possible significant deviations, which was not the case. The variability among replicated counts for CARD-FISH was checked for this lake in a previous study (Hörtnagl *et al.*, 2010) and found to be <3%.

### Statistical analysis

Before statistical analysis, the relative abundance of bacterial groups (expressed as the percentage of DAPI-stained cells) was arcsin-transformed, whereas physicochemical (except for pH and temperature) data, bacterial abundance, and absolute numbers of bacterial groups were log (*x*+1)− transformed to obtain a normal distribution. Normality was checked using the Kolmogorov–Smirnov test. Paired *t*-tests were used to compare environmental and biological parameters between the SML and the ULW. Additionally, we compared the parameters measured between 2007 and 2008 for both layers using *t*-tests. The program sigmastat (Systat Software Inc., Point Richmond, CA) was used for these tests. To reveal the relationships of environmental variables with the absolute abundance of bacterial groups, a redundancy analysis (RDA) was performed using the software canoco (Ter Braak & Šmilauer, 1998). The explanatory power of all canonical axes was checked by running 1999 unrestricted Monte Carlo permutations.

## Results

### Physicochemical parameters

**Fig. 1:**
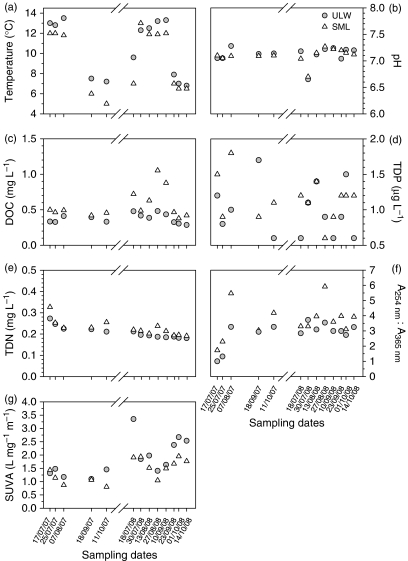
Physicochemical parameters of the SML (open triangles) and the ULW (filled circles) for 2007 and 2008 in Gossenköllesee (GKS). (a) water temperature, (b) pH, (c) DOC, (d) TDP, (e) TDN, (f) DOM absorption ratio between 254 and 365 nm, and (g) SUVA at 254 nm.

During both sampling periods, the water temperature (Fig. 1a) was significantly higher in the ULW than in the SWL (paired *t*-test: *P*<0.001). No significant differences in the pH values were detected between both layers. The pH ranged from 7.04 to 7.28, except on July 30, 2008, when a minimum value was recorded in both layers (SML: 6.70, ULW: 6.65; Fig. 1b).

The DOC concentration ranged from 0.38 to 1.05 mg L^−1^ in the SML and from 0.29 to 0.48 mg L^−1^ in the ULW. The highest DOC concentration was found in the SML on August 27 (1.05 mg L^−1^), followed by a second maximum on September 10, 2008 (0.88 mg L^−1^, Fig. 1c). The DOC concentration was significantly higher in the SML than in the ULW (paired *t*-test: *P*<0.001).

The TDP concentration in both layers was not significantly different and ranged from 0.6 to 1.8 μg L^−1^ in the SML and from 0.6 to 1.7 μg L^−1^ in the ULW (Fig. 1d). The concentration of TDN (Fig. 1e) was the highest in July 2007 (SML: 0.33 mg L^−1^ and ULW: 0.27 mg L^−1^) and the lowest in October 2008 (SML: 0.19 mg L^−1^ and ULW: 0.18 mg L^−1^) and seemed to decrease in both years from summer to autumn. The concentration of TDN was significantly higher in the SML than in the ULW (paired *t*-test: *P*<0.01) and significantly higher in 2007 compared with 2008 for both layers (*t*-test: SML *P*<0.01, ULW *P*<0.001).

The *a*_254_ : *a*_365_ ratio was the lowest on July 17, 2007 in both layers (1.72 in the SML and 1.00 in the ULW). This ratio increased steadily in 2007, but showed quite constant values in 2008 in both layers (Fig. 1f). The *a*_254_ : *a*_365_ ratio was significantly higher in the SML compared with the ULW (paired *t*-test: *P*<0.01). The absorption coefficient at 254 nm (SUVA) in the SML ranged from 0.80 to 1.95 L mg^−1^ m^−1^ and was significantly lower (paired *t*-test: *P*<0.01) than that in the ULW (range from 1.10 to 3.35 L mg^−1^ m^−1^Fig. 1g). Additionally, SUVA values were significantly higher in 2008 than in 2007 in both layers (*t*-test: SML and ULW *P*<0.01).

### Bacterial abundance and community composition

**Fig. 2:**
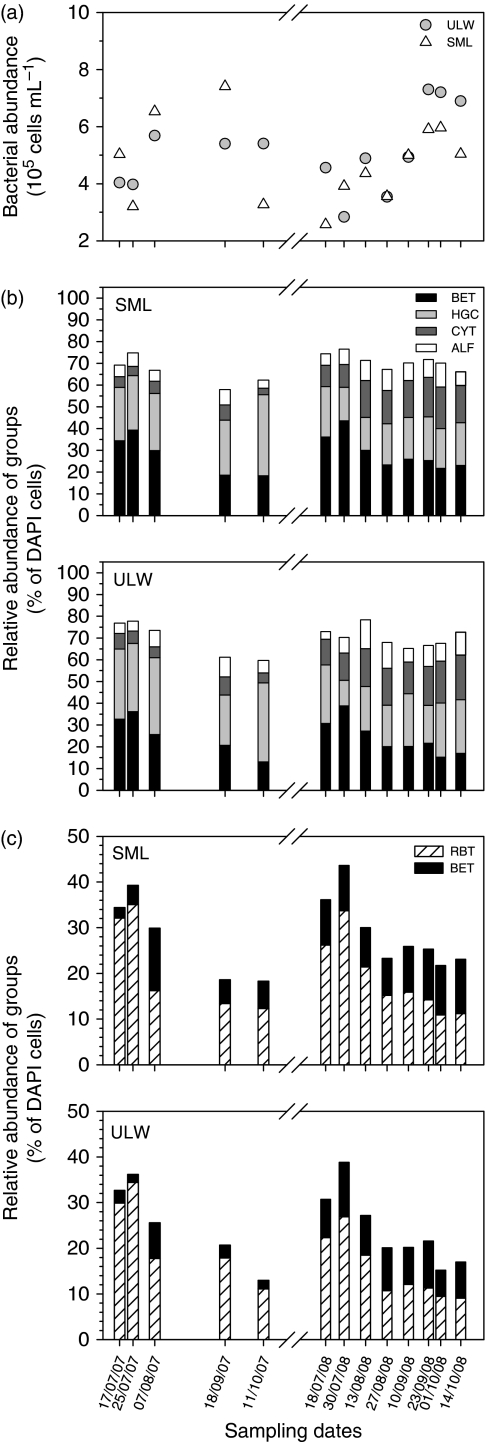
Seasonal changes in the microbial parameters of the SML and the ULW: bacterial abundance (a) and relative abundance of major bacterial groups in the SML and the ULW (b), and the R-BT subgroup of the *Betaproteobacteria* (c). Black bars represent the *Betaproteobacteria* (BET), light gray bars the *Actinobacteria* (HGC), dark gray bars the *Cytophaga*-like bacteria (CYT), white bars the *Alphaproteobacteria* (ALF), and hatched bars the R-BT subgroup of the *Betaproteobacteria*. The break on the *x*-axis separates sampling dates between 2007 and 2008.

Bacterial cell numbers differed between the SML and the ULW in most samples (Fig. 2a), but there was no consistent trend of higher bacterial numbers in either layer. Bacterial abundance ranged from 2.57 to 7.41 × 10^5^ cells mL^−1^ in the SML and from 2.83 to 7.30 × 10^5^ cells mL^−1^ in the ULW (Fig. 2a).

Between 62% and 80% of the DAPI-stained cells in both layers belonged to the domain *Bacteria*, and on average, 96% of all the cells identified as *Bacteria* were targeted with the four group-specific probes used. The bacterial community of both the SML and the ULW was dominated by *Betaproteobacteria* and *Actinobacteria* (Fig. 2b). In both layers, the relative abundance of *Betaproteobacteria* was the highest at the end of July (samples on July 25, 2007 and July 30, 2008) and decreased by 50% in the SML and by 40% in the ULW (Fig. 2b) toward the end of the sampling seasons (October 2007 and 2008). The relative abundance of *Betaproteobacteria* was slightly enriched in the SML (paired *t*-test: *P*<0.001, mean SML: 28%, mean ULW: 25%). The R-BT subgroup of *Betaproteobacteria* also showed the highest relative abundance at the end of July and decreased by about 33% in both layers till the end of October (Fig. 2c). The SML was also slightly enriched in R-BT bacteria as compared with the ULW (paired *t*-test: *P*<0.05, mean SML: 20%, mean ULW: 18%).

The relative abundance of *Actinobacteria* ranged from 15% to 37% of DAPI counts in the SML and from 11% to 36% of DAPI counts in the ULW. The relative abundance of this group was significantly different between the two layers (paired *t*-test: *P*<0.05), although the mean values were close (mean SML: 22%, mean ULW: 25%). The relative abundance of *Actinobacteria* was significantly higher in 2007 than in 2008 (*t*-test: SML and ULW *P*<0.01).

**Fig. 3:**
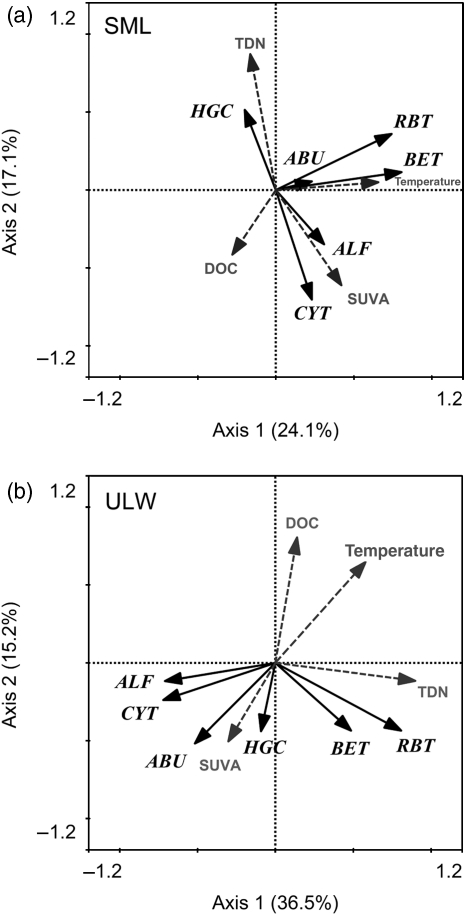
RDA biplots of selected physicochemical and biological parameters for the SML (a) and the ULW (b). The eigenvalues of the two axes are given in parentheses. DOC, dissolved organic carbon; SUVA, DOC-specific UV absorption at 254nm; TDN, total dissolved nitrogen; ABU, bacterial abundance; BET, *Betaproteobacteria*; RBT, R-BT subgroup of *Betaproteobacteria*; HGC, *Actinobacteria*; ALF, *Alphaproteobacteria*; CYT, *Cytophaga*-like bacteria. Analysis based on the absolute abundance of the different bacterial groups.

The relative abundance of *Alphaproteobacteria* was neither significantly different between layers nor between 2007 and 2008. *Cytophaga*-like bacteria were almost equally distributed in both layers. However, their relative abundance was significantly higher in 2008 than in 2007 in both layers (*t*-test: SML and ULW *P*<0.001). In both layers, the absolute abundance of *Cytophaga*-like bacteria was positively related to that of *Alphaproteobacteria* (RDA, Fig. 3a and b).

### Relationship between physicochemical parameters and bacterial community composition

The first two axes of the RDA explained 41.2% of the variability in the relative contribution of bacterial groups in the SML and 51.7% in the ULW (Fig. 3a and b). For both layers, the Monte Carlo permutation test revealed that the sum of the physicochemical variables included significantly explained the given variability of the bacterial parameters (Fig. 3a SML: *P*<0.05, Fig. 3b ULW: *P*<0.01). In the SML, a close relationship was found between water temperature and the absolute abundance of *Betaproteobacteria* and its R-BT subgroup. The *Actinobacteria* in the SML were positively related to the concentration of TDN (Fig. 3a), whereas in the ULW, they were positively related to the SUVA (Fig. 3b). In the SML and the ULW, the concentration of TDN was negatively related to *Cytophaga*-like bacteria and *Alphaproteobacteria* (Fig. 3a and b).

## Discussion

Previous studies comparing the physicochemical characteristics of the SML and the ULW have found enrichment in inorganic and organic nutrients at the air–water interface of marine (Carlson, 1982; Williams *et al.*, 1986;) and freshwater ecosystems (Södergren, 1984; Münster *et al.*, 1997;). However, little information is available on the temporal stability or trends of those parameters, due to the lack of frequently repeated measurements and the use of different sampling devices that hamper direct comparisons of the physicochemical and biological properties of the SML among similar ecosystems (Maki, 1993; Agogué*et al.*, 2004;).

In the present study, five out of seven physicochemical parameters differed significantly between the two water layers, indicating that distinct environmental conditions characterize the SML. Whereas most physicochemical parameters showed similar temporal trends in both layers (e.g. water temperature and TDN, Fig. 1a and e), others showed high variability (e.g. TDP, Fig. 1g). The consistently colder water temperature found in the SML (Fig. 1a) was probably the result of evaporation taking place at the air–water interface.

The SML was characterized by higher DOC concentrations than the ULW (Fig. 1c), which is in agreement with the results obtained in other studies (Kuznetsova *et al.*, 2004; Cunliffe *et al.*, 2009c; Hörtnagl *et al.*, 2010;). Recently, gel-like transparent exopolymer particles were found to be enriched in the SML of oceanic and estuarine waters (Wurl & Holmes, 2008; Cunliffe *et al.*, 2009b;), supporting the idea of the gelatinous nature of the SML (Sieburth, 1983) and confirming the role of the SML as a collector for surface-active organic compounds such as carbohydrates, proteins, lipids, and humic substances (Williams *et al.*, 1986; Maki, 1993; Gasparovic *et al.*, 1998;). A gel-like SML could also act as a kind of trap for organic and inorganic allochthonous material transported through the atmosphere. For example, dust depositions are known to influence the water chemistry of alpine lakes (Psenner, 1999), and airborne nutrients, especially phosphorus, can significantly contribute to the nutrient input into small lakes (Sickman *et al.*, 2003). Generally, atmospheric depositions are considered to affect the physicochemical (Maki, 1993) and biological (Hervàs & Casamayor, 2009; Hervàs *et al.*, 2009;) properties of the air–water interface. The strong enrichment in DOC (September 10, 2008) and TDP (September 10 and October 14, 2008) observed in the SML (Fig. 1c and d) was probably related to the intense deposition of Saharan dust that reached the lake one day before each sampling date. Rain water samples collected at the lake during the two Saharan dust events had high DOC (1.28 and 0.77 mg L^−1^) and TDP (3.2 and 7.4 μg L^−1^) concentrations (on September 11 and October 19, 2008, respectively, P. Hörtnagl & R. Sommaruga, unpublished data).

The significantly higher *a*_254_ : *a*_365_ ratio found in the SML is in agreement with the idea that DOM was photodegraded by the intense UV radiation found at the air–water boundary. UV radiation is known to transform DOM into molecules of a lower average molecular weight (i.e. high *a*_254_ : *a*_365_ ratio) in lake water (Bertilsson & Tranvik, 2000). This organic material was also obviously photobleached (i.e. loss of UV absorption) as indicated by the generally lower values of SUVA in the SML than in the ULW. These results are interesting because a photodegradation signature in the DOM pool is usually undetectable in this type of lake with a very low DOC concentration (<0.5 mg L^−1^), which is mainly derived from microbial processes (Sommaruga & Augustin, 2006). It is also possible that the photodegradation signature resulted from aerosol-associated DOM that has been exposed to high intensities of UV radiation during its transport through the atmosphere before it is deposited. Considering that lakes located at a high altitude are characterized by higher atmospheric deposition than those at a lower altitude (Psenner, 1999), airborne photodegraded molecules are likely to contribute occasionally to the DOM pool at the air–water boundary.

Other physicochemical parameters such as TDN showed not only a slight enrichment in the SML but also interannual differences. The significantly higher TDN concentration in 2007 was probably related to the higher total precipitation recorded during the sampling period in this year (June–October 2007: 747 mm vs. June–October 2008: 570 mm; data provided by the Tyrolean Hydroelectric Power Company). This explanation seems plausible, if we consider that the mean concentration of TDN for rain water collected at the catchment of GKS was 0.56 mg L^−1^ (P. Hörtnagl & R. Sommaruga, unpublished data).

In contrast to results from previous studies (Crawford *et al.*, 1982; Kalwasinska & Donderski, 2005; Joux *et al.*, 2006;), we did not find a consistent (only in three out of 13 sampling occasions) enrichment in the total abundance of bacteria in the SML of Gossenköllesee (Fig. 2a). Similarly, Cunliffe *et al.* (2009b) found a weakly enrichment in bacterial cell numbers in the SML of a Norwegian fjord during a mesocosm experiment. The type of sampler used to collect the SML is known to have a strong effect on the results obtained (Agogué*et al.*, 2004; Cunliffe *et al.*, 2009a;). For example, if the layer sampled is thicker than the actual SML, then, the potential enrichment in bacterial cell numbers could be masked. However, this was not the case in our study because the results from the physicochemical parameters indicated that the layer we sampled was different from the subsurface. Yet, it is difficult to pinpoint an explanation because cell abundance is affected by the balance between growth of the different bacterial members (and in the case of the SML probably also by the deposition of air-borne bacteria) and loss factors such as mortality by viruses and grazers not considered in this study. We can only speculate that photodegradation by UV radiation of mainly labile (autochthonous) DOM causes a reduction in substrate availability (i.e. organic matter becomes more recalcitrant) to bacteria as argued by Obernosterer *et al.* (1999) for marine bacterioplankton.

No obvious relationship could be found between the total bacterial abundance and the measured environmental parameters in either layer (Fig. 3a and b). Bacterial cells are known to be transported through the atmosphere (Maki, 1993; Griffin *et al.*, 2001; Amato *et al.*, 2007;) and they can be deposited at the air–water interface (Hervàs & Casamayor, 2009). Thus, one could expect that cell numbers will increase at the SML after events of high atmospheric deposition (both wet and dry). However, we could not detect an increase in cell numbers at the SML during the periods of Sahara dust deposition discussed above. This was probably because we sampled during the Saharan intrusion and a certain lag is needed to detect changes in bacterial numbers in this type of lake. Nevertheless, mineral dust particles were clearly visible in the DAPI preparations of the SML at that time (data not shown).

In both layers, the bacterial community was dominated by two common freshwater groups, namely, *Betaproteobacteria* and *Actinobacteria* (Fig. 2b). Whereas *Actinobacteria* were slightly enriched in the ULW, *Betaproteobacteria* and its R-BT subgroup were so in the SML. These findings correspond with the results of our previous work, where a similar enrichment of *Actinobacteria* in the ULW and of *Betaproteobacteria* in the SML was found for six mountain lakes (Hörtnagl *et al.*, 2010). Both *Betaproteobacteria* and *Actinobacteria* are ubiquitous groups in freshwater lakes (Methé*et al.*, 1998; Glöckner *et al.*, 2000; Zwart *et al.*, 2002;) and are numerically important in mountain lakes (Warnecke *et al.*, 2005). Members of the *Betaproteobacteria* have been found in extreme and remote environments (Amato *et al.*, 2007; Fierer *et al.*, 2008; Sommaruga & Casamayor, 2009;) and are known to respond very rapidly to organic and inorganic nutrient enrichments (Pérez & Sommaruga, 2006; Šimek *et al.*, 2006;). The dominance of *Betaproteobacteria* and its R-BT subgroup at the SML might be the result of a high colonization potential by members of this group. In fact, bacteria of the phylogenetically narrow R-BT subgroup, which are widespread in different freshwater habitats (Šimek *et al.*, 2010), are among the fastest-growing freshwater bacteria known (Šimek *et al.*, 2006). Furthermore, Hervàs *et al.* (2009) and Hervàs & Casamayor (2009) reported a high similarity between neustonic and airborne *Betaproteobacteria*, as well as a high viability and colonization potential for numerous members of the *Betaproteobacteria*.

The results presented here depict the SML of a transparent high mountain lake as a distinct microhabitat that seems to favor the colonization by specific groups of bacteria. Whereas the quantitative bacterial community composition of the SML differed slightly from that of the ULW, the absolute abundance of the different bacterial groups in the two layers seems to be associated with different environmental factors (Fig. 3). In particular, one of the dominant bacterial groups (i.e. *Betaproteobacteria*) seems to be influenced by water temperature in the SML, but not in the ULW. Interestingly, temporal changes in the bacterial community composition followed similar trends in both layers, suggesting the existence of other overriding factors not considered in our study. Overall, our results lead to several questions such as how successful air-borne bacteria are as colonizers and what factors control bacterioneuston abundance in lakes.

## Statement

Re-use of this article is permitted in accordance with the Terms and Conditions set out at http://www3.interscience.wiley.com/authorresources/onlineopen.html
